# Remote Inflammatory Preconditioning Alleviates Lipopolysaccharide-Induced Acute Lung Injury via Inhibition of Intrinsic Apoptosis in Rats

**DOI:** 10.1155/2021/1125199

**Published:** 2021-09-20

**Authors:** Yong Liu, Jiahang Xu, Liang Zhao, Jing Cheng, Baojun Chen

**Affiliations:** Department of Thoracic Surgery, The Central Hospital of Wuhan, Tongji Medical College, Huazhong University of Science and Technology, Wuhan 430011, China

## Abstract

**Background:**

Acute lung injury (ALI) always leads to severe inflammation. As inflammation and oxidative stress are the common pathological basis of endotoxin-induced inflammatory injury and ischemic reperfusion injury (IRI), we speculate that remote ischemic preconditioning (RIPC) can be protective for ALI when used as remote inflammatory preconditioning (RInPC).

**Method:**

A total of 21 Sprague-Dawley rats were used for the animal experiments. Eighteen rats were equally and randomly divided into the control (NS injection), LPS (LPS injection), and RInPC groups. The RInPC was performed prior to the LPS injection via tourniquet blockage of blood flow to the right hind limb and adopted three cycles of 5 min tying followed by 5 min untying. Animals were sacrificed 24 hours later. There were 2 rats in the LPS group and 1 in the RInPC group who died before the end of the experiment. Supplementary experiments in the LPS and RInPC groups were conducted to ensure that 6 animals in each group reached the end of the experiment.

**Results:**

In the present study, we demonstrated that the RInPC significantly attenuated the LPS-induced ALI in rats. Apoptotic cells were reduced significantly by the RInPC, with the simultaneous improvement of apoptosis-related proteins. Reduction of MPO and MDA and increasing of SOD activity were found significantly improved by the RInPC. Increasing of TNF-*α*, IL-1*β*, and IL-6 induced by the LPS was inhibited, while IL-10 was significantly increased by RInPC, compared to the LPS group.

**Conclusion:**

RInPC could inhibit inflammation and attenuate oxidative stress, thereby reducing intrinsic apoptosis and providing lung protection in the LPS-induced ALI in rats.

## 1. Introduction

Acute lung injury (ALI) is a life-threatening parenchymal lung disease caused by various pathogenic factors. The ALI is characterized by hypoxemia, lung gas and blood barrier damage, bilateral pulmonary inflammatory infiltration, and noncardiogenic interstitial edema. It often progresses to acute respiratory distress syndrome (ARDS) and requires mechanical ventilation. Uncontrolled inflammation is the main cause of death, with a mortality rate of over 30% [1]. At present, the treatment for ALI/ARDS is mainly supportive, and novel therapeutic strategies are urgently needed.

Sepsis is the most common cause of ALI. Lipopolysaccharide (LPS), the endotoxin derived from the outer membrane of Gram-negative bacteria, which is believed to be one of the most frequent triggers of sepsis, is a powerful causative agent of systemic inflammation. The LPS can directly damage the alveolar-capillary barrier, lung epithelial cells, and pulmonary vascular endothelial cells [2]. Alveolar macrophages (AM) activated by LPS can release cytokines such as TNF-*α* and IL-1*β* to initiate the inflammatory cascade, producing a large number of inflammatory mediators and factors, and reactive oxygen species (ROS). The ROS can destroy the gas and blood barrier by damaging pulmonary vascular endothelial cells and alveolar epithelial cells, increasing their permeability, and causing pulmonary edema; it can also upregulate the expression of inflammatory factors and induce inflammation [3]. It has been elucidated that several different forms of programmed cell death (PCD), including autophagy, apoptosis, and pyroptosis, have been correlated with the LPS-induced ALI in rat models [4–6].

Pyroptosis is triggered in response to infection. The LPS has been reported to directly stimulate the activation of caspase-11, which cleaves gasdermin D (GSDMD) resulting in membrane rupture and cell lysis in rodents [7]. The innate immune response can be activated by LPS through the activation of TLR4 receptors [8], leading to the transcription of MyD88-dependent genes, which encode proinflammatory cytokines including inactive proforms of IL-1*β* and inflammasome components [9]. Multiple studies elucidated the role of the Fas/FasL system in the extrinsic epithelial apoptosis in LPS-induced ALI [6]. DNA damage, hypoxia, and metabolic stress can induce intrinsic apoptosis, which begins with mitochondrial outer membrane permeabilization (MOMP) and leads to the release of mitochondrial proteins into the cytosol [10]. The ROS may stimulate the cell death pathways and trigger inflammation, resulting in inflammasome activation, pyroptosis [11], and intrinsic apoptosis.

Ischemia-reperfusion injury (IRI) refers to the irreversible tissue damage caused by insufficient oxygen supply following tissue ischemia and subsequent restoration of blood supply. Oxidative stress, inflammation, and calcium ion overload were involved with the ischemia-reperfusion injury [12]. Ischemic preconditioning (IPC) is currently known as an effective protection strategy against the IRI. Remote IPC (RIPC) can be used to offer a protective effect to the target organ by transient ischemic interventions in organs or tissues far away from the target. In previous studies, the protective effect of the RIPC against myocardial IRI and cerebral IRI has been demonstrated in rat models [13, 14]. Its protective mechanism was related to the reduction of oxidative stress and the alleviation of intrinsic apoptosis.

Based on the results from this study, we speculate that the RIPC can also be used as a novel protective strategy in LPS-induced ALI via alleviating intrinsic apoptosis. To facilitate the distinction, RInPC, a short-term ischemic intervention in organs or tissues far away from the target organ before inflammation occurs, is termed to stand for remote inflammatory preconditioning, which is distinguished from RIPC. The LPS-induced ALI rat models were used with the RInPC during the preinflammatory stage to verify this hypothesis and explore its intrinsic apoptosis-related mechanisms.

## 2. Materials and Methods

### 2.1. Ethics Statement

All animals were taken care of and treated in agreement with the Animal Research: Reporting of In Vivo Experiments (ARRIVE) guidelines for this study. Further, all animal procedures were performed following the guidelines of Institutional Animal Care. The ethics approval has been obtained from the Ethics Committee of the Central Hospital of Wuhan affiliated to Tongji Medical College, Huazhong University of Science and Technology, before the onset of the study.

### 2.2. Animals

A total of 21 Sprague-Dawley rats weighing 250 to 270 g were obtained from the Beijing Vital River Laboratory Animal Technology Co., Ltd. (Certificate Number: SCXK-2003-001; Beijing, China). The animal experiment occurred at the animal experimental center of the Biofavor Biotech Company in Wuhan, Hubei, China. Animals were maintained in an air-conditioned atmosphere at 25°C with a 12-hour light-dark cycle exposure and were provided with free access to pelleted food and ad libitum water. After a one-week acclimation, the animals were randomly assigned into three groups, six rats per group. The first group was maintained as the control. The second group (LPS group) had the LPS intravenous injection. The third group (RInPC group) was treated the same as the LPS group with additional 30 minutes of remote stimuli before the LPS injection. There were 2 rats in the LPS group and 1 rat in the RInPC group that died before the end of the experiment. Supplementary experiments for the LPS and RInPC groups were conducted to include 6 animals that reached the end of the experiment in each group.

### 2.3. Drugs

The LPS (O127: B8; Sigma, St. Louis, MO, USA) used in this study was derived from Escherichia coli (O127) endotoxin, and it was dissolved in sterile saline.

### 2.4. Experimental Protocol

The animal model of LPS-induced ALI was developed with some modifications as described by Hagiwara et al. [15]. Briefly, the rat model was created by injection of LPS (5 mg/kg) via the tail vein. The same volume of normal saline (NS) was administered to the animals in the control group through the same route. All animals were injected intravenously under ether inhalation anesthesia.

The RInPC was performed for 30 minutes ahead of the LPS injection via tourniquet blockage of blood flow to the right hind limb and adopted three cycles of 5 min tying followed by 5 min of untying. Circulatory arrest in the limbs was identified by observing the empurpled limb skin and confirmed using a vascular Doppler. This method has been developed and standardized in a previous study [16].

Twenty-four hours after the injection, the animals were sacrificed following heart blood sampling under overanesthesia. The serum was separated by centrifugation of the blood sample at 3000 g for 15 minutes. Lung samples were collected with inflation after the chest was opened. The left lungs were used to measure the wet/dry ratio. The right upper lungs were stored in 4% paraformaldehyde for histological studies. And the right lower lungs were stored at −80°C for biochemical assay and protein analysis by western blotting.

### 2.5. Histology and Morphology

Complete random cross-sections of the rat lungs were fixed in 4% neutral phosphate-buffered formaldehyde, embedded in paraffin, sectioned (5 *μ*m), and stained with hematoxylin and eosin (H&E). The sections were viewed by an experienced morphologist who knew nothing about the sample identity. Ten randomly chosen microscopic fields (×200) were viewed for each lung sample, and all 6 samples were viewed for each animal group. Histological evidence suggesting ALI was also evaluated by a blinded investigator according to Hofbauer and colleagues' method [17]. In which, alveolar membrane thickness and cellularity were evaluated by estimating the fraction of the microscopic field occupied by the parenchymal tissue as opposed to the empty alveolar spaces. The average values of ALI were represented by a histological index of quantitative assessment (IQA) using the following criteria. Samples were graded from normal to severe, which was expressed by 0 (<15% of the space occupied by tissue and >85% by alveolar space), 1+ (15%-25% occupied by tissue and 75%-85% by alveolar space), 2+ (25%-50% occupied by tissue and 50%-75% by alveolar space), 3+ (50%-75% occupied by tissue and 25%-50% by alveolar space), and 4+ (75%-100% occupied by tissue and 0%–25% by alveolar space).

### 2.6. Lung Wet-to-Dry Weight Ratio Measurement

To assess tissue edema, the weight of rat lungs (six lungs per group) was measured, followed by a drying step of the lungs in an oven at 80°C for 48 h until the weight of the samples became constant. Then, the lung wet-to-dry weight ratio was calculated.

### 2.7. Assay of Serum Lactate Acid

Serum lactate measurement was performed in all groups using a lactate assay kit (Nanjing Jiancheng Bioengineering Institute, Nanjing, Jiangsu, China), according to the manufacturer's instructions.

### 2.8. Enzyme-Linked Immunosorbent Assay (ELISA)

The levels of TNF-*α*, IL-1*β*, IL-6, and IL-10 in serum were detected using the specific mouse or human ELISA kits (Elabscience Biotechnology Co. Ltd., Wuhan, Hubei, China). The optical density was measured at 450/540 nm wavelength using an automated ELISA reader (Flexstation3, Molecular Devices, LLC, Sunnyvale, CA, USA). All standards and samples were run in triplicate.

### 2.9. Assays of Malondialdehyde (MDA), Myeloperoxidase (MPO), and Superoxide Dismutase (SOD)

These three oxidative stress indicators were detected in serum, as previously reported by using commercial assay kits (Nanjing Jiancheng Bioengineering Institute), according to the manufacturer's instructions [18]. The unit of measurement for MDA was nmol per milligram of protein. MPO and SOD activities were expressed as units per milligram of protein.

### 2.10. Terminal Deoxynucleotidyl Transferase-Mediated dUTP Nick End Labeling (TUNEL) Assay

The TUNEL technique was carried out using the “In Situ Cell Death Detection Kit.” Briefly, the lung sections on the microscopic slides were dewaxed and incubated with proteinase K. Then, the slides were stained using a TUNEL kit (Biovision Inc., Mountain View, CA, USA), according to the manufacturer's instructions. Subsequently, the slides were examined under a fluorescence microscope (Olympus BX53, Olympus, Japan). Images were captured to determine the percentage of positive cells and intensity of staining and then used to calculate the percentage of positive nuclei in three representative areas from three samples per group as the apoptotic index for statistical analysis.

### 2.11. Western Blotting Analysis

The right lower lung specimens (approximately 100 mg each) were dissected out and stored at -80°C. The protein expressions of Bcl-2, Bax, Cyt-c, AIF, caspase-3, cleaved caspase-3, caspase-9, and cleaved caspase-9 in the lung were detected by western blotting analysis, which was described in the literature [19]. Briefly, the protein concentration was determined by the Bicinchoninic Acid (BCA) method. The protein sample was boiled and denatured; then, SDS-PAGE gel electrophoresis was performed. The protein was transferred onto the nitrocellulose membrane. Next, the proteins were blocked with 5% skim milk at 37°C for 1 h. The membranes were incubated overnight at 4°C with diluted primary antibody and GADPH primary antibody (1 : 1000). The next day, the membrane was washed three times with TBST and incubated with a secondary antibody diluted with the blocking solution at 37°C for 2 hours. The enhanced chemiluminescence (ECL) was developed, and the protein bands were photographed after washing. The integral optical density (IOD) of each target band was determined using Bandscan 5.0 software (*Bio Marin Pharmaceutical*, San Rafael, CA, USA). The expressions of the target proteins were normalized by the ratio of integrated optical density (IOD) of proteins to the IOD of GADPH. The expressions of Cyt-c and AIF in the mitochondria were normalized by the ratio of the IOD of proteins to the IOD of COX4.

### 2.12. Statistical Analysis

The significant differences were calculated using one-way ANOVA among multiple groups with the Prism 8.0 software (GraphPad Software, Inc., San Diego, CA, USA). Results were expressed as means ± standard deviation (SD). Values are shown using a column diagram. *P* < 0.05 was considered significant.

## 3. Results

### 3.1. RInPC Attenuated the LPS-Induced ALI in Rats

**Figure 1:**
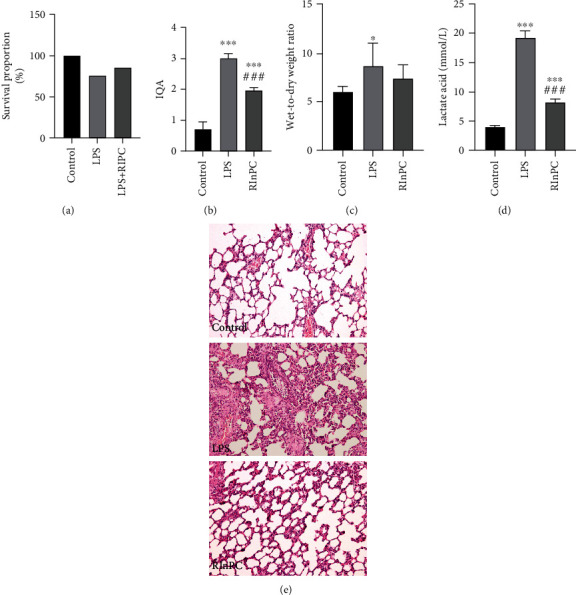
RInPC attenuated LPS-induced ALI in rats. (a) Comparison of survival proportion among three groups. (b) Comparison of ALI represented by IQA between each group. (c) Comparison of wet/dry ratio between each group. (d) Comparison of lactic acid in the serum of each group. (e) Histological changes of lung tissue in each group (H&E; 200x). Control: control group; LPS: LPS injury group; RInPC: LPS+RInPC group. In comparison, the LPS injury group showed diffuse edema in alveolar spaces and interstitium of the lung, hemorrhage, severe inflammatory cell infiltration and serous exudation in the alveolar space, and thickened interlobular septa. These changes were significantly mitigated in the RInPC group. ^∗^*P* < 0.05; ^∗∗^*P* < 0.01; ^∗∗∗^*P* < 0.001 vs. control group; ^#^*P* < 0.05; ^##^*P* < 0.01; ^###^*P* < 0.001 vs. LPS group (*n* = 6); IQA: index of quantitative assessment.

The survival percentages of the three groups of models were 100% (6/6), 75.0% (6/8), and 85.7% (6/7), respectively (Figure 1(a)). Histological evaluations of lung tissue changes by H&E staining were compared among the three groups. Similar to the description by Du et al. [20], the morphology in the control group was normal with no fluid in the alveolar space. No evidence of inflammatory cell infiltration or hemorrhage on the alveolar wall was found. Diffuse edema in alveolar spaces, inflammatory cell infiltration, and thickened interlobular septa were found in both the LPS and RInPC groups. A significantly higher ALI score represented by IQA was observed in the LPS group compared to the others. The IQA score of the RInPC group was significantly lower than that of the LPS group (control vs. RInPC vs. LPS: 0.71 ± 0.24 vs. 1.96 ± 0.10 vs. 3.00 ± 0.16, *P* < 0.001) (Figures 1(b) and 1(e)).

The wet/dry lung weight ratio was significantly increased in the LPS group (8.66 ± 2.34 vs. 6.02 ± 0.60, *P* < 0.05) compared to the control group. The wet/dry ratio in the RInPC group was between the control and LPS groups, without any significant differences (Figure 1(c)).

The value of lactate acid in both the LPS group and the RInPC group was significantly increased compared to the control, while the value in the RInPC group was significantly lower than that in the LPS group. The values of the three groups were 3.98 ± 0.33, 19.33 ± 1.03, and 8.22 ± 0.51, respectively (*P* < 0.001) (Figure 1(d)).

### 3.2. RInPC Prevented Apoptosis via an Intrinsic Pathway in LPS-Induced ALI in Rats

**Figure 2:**
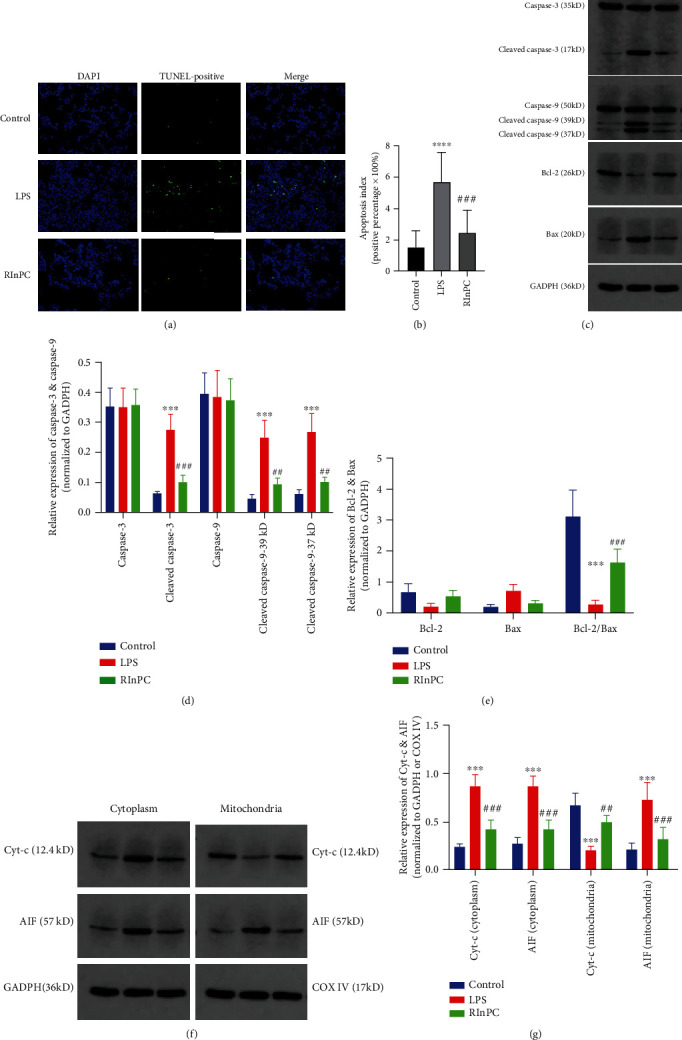
RInPC prevented apoptosis via an intrinsic pathway in LPS-induced ALI in rats. (a) Fluorescent images of the TUNEL results under a fluorescence microscope in ALI rats induced by LPS (original magnification: 400x). (b) Comparison of apoptosis index between each group. (c) WB bands of caspase-3, cleaved caspase-3, caspase-9, cleaved caspase-9, Bcl-2, and Bax. (d) Comparison of caspase-3, cleaved caspase-3, caspase-9, and cleaved caspase-9 between each group. (e) Comparison of Bcl-2, Bax, and Bcl-2/Bax ratio between each group. (f) WB bands of Cyt-c and AIF expressed in the cytoplasm and mitochondria. (g) Comparison of Cyt-c and AIF in the cytoplasm and mitochondria between each group. ^∗∗∗^*P* < 0.001; ^∗∗∗∗^*P* < 0.0001 vs. control; ^##^*P* < 0.01; ^###^*P* < 0.001 vs. LPS (*n* = 3). DAPI: 4′,6-diamidino-2-phenylindole; WB: western blot; Cyt-c: cytochrome c; AIF: apoptosis-inducing factor; GAPDH: glyceraldehyde 3-phosphate dehydrogenase; COX4: cytochrome c oxidase subunit 4.

To determine the protective effects of the RInPC against LPS-induced apoptosis, TUNEL was performed. In vivo, LPS-challenged animals exhibited a significant increase in green fluorescence apoptotic cells, which was significantly reduced by the RInPC (Figures 2(a) and 2(b)).

Although the values of both caspase-3 and caspase-9 were not changed in the lung specimen, cleaved caspase-3 and cleaved caspase-9 were upregulated significantly (*P* < 0.001 compared with the control group). The RInPC inhibited the LPS-induced upregulation of cleaved caspase-3 and cleaved caspase-9 (*P* < 0.01 compared with the LPS group) (Figures 2(c) and 2(d)).

The intrinsic pathway of apoptosis, which means mitochondrial-dependent apoptosis, is mediated through the release of cytochrome c (Cyt-c) and apoptosis-inducing factor (AIF), leading to ultimately caspase activation. In the present study, significantly increased Cyt-c in the cytoplasm and decreased Cyt-c in the mitochondria were observed (*P* < 0.001 compared with the control group), which was alleviated by the RInPC (*P* < 0.001 compared with the LPS group). Simultaneously, increased AIF both in cytoplasm and mitochondria were observed (*P* < 0.001 compared with the control group), which was also alleviated by the process of RInPC (*P* < 0.001 compared with the LPS group) (Figures 2(f) and 2(g)).

Additionally, the present study investigated the changes in the expression levels of the Bcl-2 family proteins (Bcl-2 and Bax) in lung tissue. The LPS injection resulted in the downregulation of the antiapoptotic protein Bcl-2 and upregulation of the proapoptotic protein Bax. Although no significant differences of Bcl-2 and Bax were observed among the three groups, a significantly decreased Bcl-2/Bax ratio was observed (*P* < 0.001 compared with the control group), and the RInPC prevented this decreased ratio (*P* < 0.001 compared with the LPS group). These results indicated that intravenous administration of LPS induced lung cell apoptosis, which was significantly alleviated by the treatment with the RInPC (Figures 2(c) and 2(e)).

### 3.3. RInPC Palliated the Oxidative Stress in Lung Induced by LPS Injection

**Figure 3:**
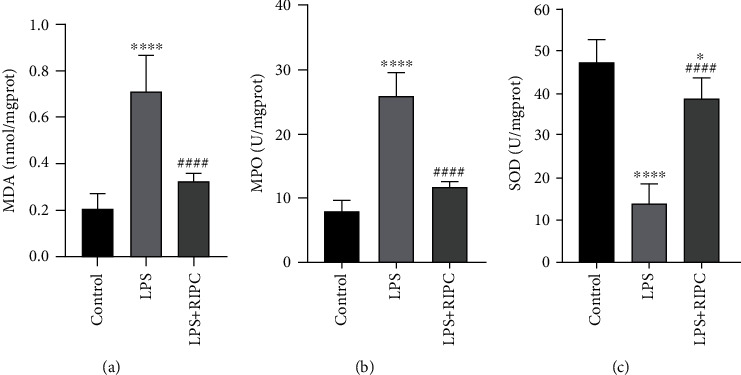
The RInPC palliated the oxidative stress in the lung induced by the LPS. (a) Comparison of MDA between each group. (b) Comparison of MPO between each group. (c) Comparison of SOD between each group. ^∗^*P* < 0.05; ^∗∗∗∗^*P* < 0.0001 vs. control group; ^####^*P* < 0.0001 vs. LPS group (*n* = 6). MPO: myeloperoxidase; MDA: malondialdehyde; SOD: superoxide dismutase.

To determine the antioxidative effects of the RInPC against LPS-induced ALI in rats, the MDA, MPO, and SOD levels in serum were measured. The LPS injection induced a 2.30-fold elevation of MDA level, a 2.13-fold elevation of MPO activity, and a 71.0% reduction of SOD activity, respectively, compared with the control group. In contrast, these oxidative markers were significantly improved by the RInPC in the LPS-injected rats. The MDA and MPO were reduced to levels close to the control group, and SOD was elevated to a level which was almost 84.5% of the control group (Figures 3(a)–3(c)).

### 3.4. The RInPC Reduced Proinflammatory Cytokine Secretion Induced by LPS

**Figure 4:**
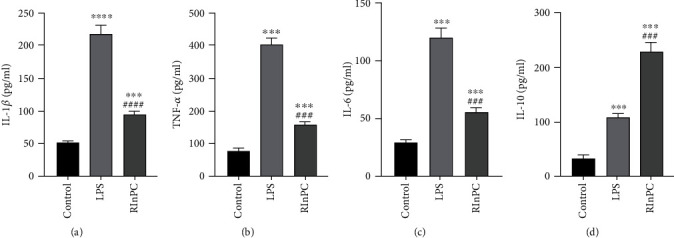
The RInPC reduced proinflammatory cytokine secretion induced by the LPS. (a) Comparison of TNF-*α* between each group. (b) Comparison of IL-1*β* between each group. (c) Comparison of IL-6 between each group. (d) Comparison of IL-10 between each group. ^∗∗∗^*P* < 0.001; ^∗∗∗∗^*P* < 0.0001 vs. control group; ^###^*P* < 0.001; ^####^*P* < 0.0001 vs. LPS group (*n* = 6). TNF-*α*: tumor necrosis factor-alpha; IL-1*β*: interleukin-1*β*; IL-6: interleukin-6; IL-10: interleukin-10.

To investigate the anti-inflammatory effects of the RInPC in the lung of LPS-intoxicated rats, TNF-*α*, IL-1*β*, IL-6, and IL-10 levels were measured. The LPS injection induced a 4.22-, 3.28-, 3.11-, and 2.20-fold elevation of TNF-*α*, IL-1*β*, IL-6, and IL-10 levels, respectively, compared with the control group. Conversely, proinflammatory cytokines were significantly improved by the RInPC of LPS-injected rats. The TNF-*α*, IL-1*β*, and IL-6 levels were improved to a level which was less than half of the level in the LPS group, with a significant increase of anti-inflammatory cytokine, IL-10, to a level which was more than 2-fold of the level in the LPS group (Figures 4(a)–4(d)).

## 4. Discussion

In this study, we demonstrated that the RInPC significantly attenuated the LPS-induced ALI in rats, possibly via an inhibition of intrinsic apoptosis, associated with reductions in both oxidative stress and proinflammatory cytokines. Although investigations on the inhibition of pyroptosis [7] and extrinsic apoptosis [6] in the LPS-induced ALI have been reported previously, we have not found a similar research result about intrinsic apoptosis and LPS-induced ALI.

Gram-negative bacteria have been associated with approximately 50% of infectious ALI, usually from pneumonia or sepsis [21]. The LPS, as a common endotoxin, is critical for organ dysfunction and mortality associated with severe Gram-negative infections [22, 23]. It has been well established that intravenous administration of LPS can induce a model of ALI [24–26].

The RIPC was originally one of the strategies to alleviate organ IRI. It has been reported to exert a protective effect against ischemia/reperfusion injury in rat hearts, brains, and other organs, which may be associated with inhibiting the opening of mPTP [27, 28]. It has also been demonstrated to regulate the human myocardial apoptosis and inflammation, which is associated with the caspase cascade [29]. The protective mechanism has been known to be related to inhibiting inflammation, reducing oxidative stress, and reducing intrinsic apoptosis. Although different mechanisms about cell death are involved between IRI and ALI, the RInPC, which we abbreviated to stand for remote inflammatory preconditioning, was suspected to be protective in this rats' ALI model induced by LPS based on the effect related to inhibition of intrinsic apoptosis.

The animal model was established through intravenous injection of LPS (5 mg/kg) in the present study, based on a previous report [30, 31]. It was observed with significant lung injury and dysfunction following LPS administration, evidenced by the deterioration of histopathology, increased wet/dry weight ratio of the lung, and elevated lactate acid in serum, which is consistent with the other studies [26, 31–33]. The ALI in rats was attenuated by the performance of RInPC, which was reflected by improved histopathological changes and decreased wet/dry ratio and lactate acid in serum compared to the LPS group. Although the value of PaO_2_/FiO_2_ was not clarified, lactate acid has been certified to be an indicator of anoxemia of the organ, especially in lung injury. The lactate level has long been used as a marker of resuscitation, for risk stratification, and as a mortality prediction tool in sepsis with the commonly held belief that elevated lactate levels in sepsis occur as a consequence of anaerobic metabolism from tissue malperfusion [34]. Cytopathic hypoxia and direct mitochondrial impairment have been proposed as a cause of hyperlactemia, although the exact mechanism remains incompletely understood [35].

Through TUNEL detection, it was confirmed that the apoptosis of lung cells existed in the ALI model, and the RInPC significantly reduced the occurrence of apoptosis. Intrinsic apoptosis, mitochondrial-dependent apoptosis, was activated through the mitochondrial release of Cyt-c, AIF, and Smac [36]. When Cyt-c entered the cytoplasm, the apoptosome assembly was released from the apoptotic protease-activating factor 1, ATP, and procaspase-9, leading to cellular apoptosis via the activation of caspase-3 and caspase-7 [37]. To further elucidate the present hypothesis of intrinsic apoptosis, the Cyt-c and AIF levels in the cytoplasm and mitochondria were measured. The RInPC was demonstrated to improve the mitochondrial release of the Cyt-c into the cytoplasm and thus the expression of AIF.

The apoptosis-related proteins play pivotal roles in apoptosis. The caspase-3 and caspase-9 are activated and regulated by the apoptotic pathway mediated by the Bcl-2/Bax ratio [38, 39]. The present results demonstrated that the RInPC significantly downregulated the expression of caspase-9 and caspase-3, the proapoptosis protein, and the executive protein of apoptosis in vivo. In addition, the antiapoptosis protein Bcl-2 and the proapoptosis protein Bax, both involved in the regulation of the opening of mitochondrial permeability transition pore (mPTP), were also analyzed. The values indicated that the RInPC could attenuate the opening of mPTP through regulation of the Bcl-2/Bax ratio to inhibit the release of Cyt-c and AIF.

To explore the ability of the RInPC in regulating oxidative stress, we tested the contents of MDA and MPO and the activity of SOD. The MDA indirectly reflects the severity of the cells being attacked by free radicals. The MPO activity is an indicator of neutrophil infiltration in the lung. The SOD is an important oxygen-free radical scavenger [40]. It was shown that the LPS injection caused an increase in MDA production, MPO secretion, and SOD consumption in rats, suggesting an induced imbalance of oxidative stress. It was also demonstrated that the RInPC was found to be a good alleviator for the imbalance of oxidative stress induced by the LPS.

In this rat model of LPS-induced ALI, it was observed that the secretion of proinflammatory cytokines, including TNF-*α*, IL-*β*, and IL-6, as well as the anti-inflammatory cytokine IL-10, was all increased significantly after the administration of the LPS, consistent with previous studies [15, 41]. Monocytes and macrophages secrete cytokines such as TNF-*α*, IL-*β*, and IL-6 during the early stage of the inflammatory response when activated by the LPS, which play an important role in the occurrence and development of ALI/ARDS [32, 42, 43]. TNF-*α* is a primary mediator of inflammation [32, 43]. The IL-1*β* also appears in the early stage of ALI and cooperates with the TNF-*α* to promote an inflammatory response. Levels of the IL-6 positively correlate with mortality in experimental models of sepsis. Measuring the IL-6 levels in at-risk patients can accurately predict individuals who are at significant risk of death as a result of sepsis [44]. The IL-10 inhibits the expression of proinflammatory cytokines, chemokines, and chemokine receptors as well as allergen tolerance in allergen-specific immunotherapy [42]. The RInPC significantly suppressed the secretion of TNF-*α*, IL-*β*, and IL-6, promoting the secretion of IL-10, which suggested that the RInPC could reduce the inflammatory response in this ALI model.

Pyroptosis exerts a cell type-dependent role in inflammation and immunity. The caspase-11-dependent noncanonical pyroptosis was activated by cytosolic LPS from invading Gram-negative bacteria in macrophages, monocytes, or other cells in rodent animals [7]. As intrinsic apoptosis is always induced by DNA damage, hypoxia, and metabolic stress; we speculated that the intrinsic apoptosis may have been secondary to noncanonical pyroptosis in the LPS-induced ALI models, and further research is needed.

Some limitations in this study exist because of the experimental design. First of all, the protective effect of the RInPC on ALI was discussed only in rodent in vivo models. To determine whether there is a similar effect on other animals or humans, more elucidations are warranted. The second is that the in vitro experiments have not been applied to explore whether cells treated with hypoxia and reoxygenation can better resist the endotoxin damage. Another one is that the wet/dry ratio was showed to have a significant difference between the control group and the LPS group, but that of the RInPC group was without any significant differences compared to the other two groups. Measurement of the protein level in BALF may be a better choice in future experiments. The last one is that the study showing some protective effects of RInPC on the LPS-induced ALI correlated with the intrinsic apoptosis is still observational. The mechanism mediating this protection has not been fully investigated.

## 5. Conclusion

In the present study, the RInPC inhibited the inflammatory response and attenuated the oxidative stress, thereby reducing intrinsic apoptosis and ultimately providing lung protection in the LPS-induced ALI model in rats. If a similar effect could be found in other animal models or human beings, we may get a new strategy to fight against ALI and ARDS.

## Data Availability

The datasets used and/or analyzed during the current study are available from the corresponding authors on reasonable request.
